# Author response to letter to the editor: “impact of the COVID-19 pandemic on brain death detection in German hospitals: a state-wide analysis of health data”

**DOI:** 10.1186/s42466-025-00412-0

**Published:** 2025-09-25

**Authors:** Daniela Schoene, Kristian Barlinn

**Affiliations:** Department of Neurology, Faculty of Medicine, University Hospital Carl Gustav Carus, Technische Universität Dresden, Dresden, Germany

**Keywords:** COVID-19, COVID-19 pandemic, Brain death, Brain death detection, German hospitals

We appreciate Calixto Machado`s insightful comments on our recent study titled “Impact of the COVID-19 pandemic on brain death detection in German hospitals: a state-wide analysis of health data”. The points mentioned, particularly regarding diagnostic challenges, ethical burden and structural limitations, reflect the real-world challenges faced by intensive care teams in Germany and around the world during the pandemic.

Our study focused on quantitative trends, but we acknowledge that these data reflect clinical complexities that go beyond what quantitative analysis alone can capture. The term ‘detection deficit’ was adopted based on prior national analyses (Schulte et al., 2018) and was not intended to imply organizational failure or a lack of physician commitment. Rather, our aim was to describe changes in brain death (BD) determination rates throughout the pandemic.

We fully recognize that infection prevention measures, severe pulmonary compromise and circulatory instability may have made BD determination medically unsafe in certain cases. These factors are essential for understanding the shifts observed during the pandemic. Nonetheless, we would like to emphasize that BD determination remained technically feasible in many cases, although these factors may have contributed to delays or precluded completion in some cases. Further analysis could help determine how often COVID-19–related factors directly impacted BD determination.

We also acknowledge the author’s call for greater structural flexibility. In Germany, the absence of DCD pathways and the opt-in consent model limit the system’s adaptability. While flexible approaches—such as temporary protocol modifications under ethical oversight—are conceptually valuable, their application in Germany would require careful legal and societal debate. Public sensitivity around brain death and organ donation underscores the need for diagnostic clarity, consistency, and trust.

In this context, innovative tools such as telemedicine - though currently limited in applicability for BD determination in Germany - may offer potential for strengthening diagnostic capacity, especially in hospitals with limited neurological expertise. International models suggest that tele-neurology consultations can support standardization and timely decision-making in complex clinical settings. While regulatory and infrastructural barriers remain, the pandemic has underscored the importance of exploring such tools to support the resilience of critical care and transplant systems.

We further acknowledge the emotional and ethical challenges described by the author. As our study focused on secondary quantitative data, we were unable to directly assess psychosocial dimensions. Given the experience of the COVID-19 pandemic, we believe future research should investigate how psychological stress influences clinical decision-making, particularly in the context of BD determination. Such studies may help inform guidelines and support structures to protect healthcare professionals´ mental health and ensure ethical decision-making during crises.

In conclusion, we thank the author for enriching this discussion. His reflections underscore that BD determination is not only a medical and legal act, but also a deeply human one, shaped by clinical context, emotional realities and systemic pressures. Future discussions on resilience must carefully balance adaptability with diagnostic accuracy and ethical responsibility.

## Data Availability

Not applicable.

